# Humans and Great Apes Cohabiting the Forest Ecosystem in Central African Republic Harbour the Same Hookworms

**DOI:** 10.1371/journal.pntd.0002715

**Published:** 2014-03-20

**Authors:** Hideo Hasegawa, David Modrý, Masahiro Kitagawa, Kathryn A. Shutt, Angelique Todd, Barbora Kalousová, Ilona Profousová, Klára J. Petrželková

**Affiliations:** 1 Department of Biology, Oita University, Yufu, Oita, Japan; 2 Department of Pathology and Parasitology, Faculty of Veterinary Medicine, University of Veterinary and Pharmaceutical Sciences, Brno, Czech Republic; 3 Central European Institute of Technology, University of Veterinary and Pharmaceutical Sciences, Brno, Czech Republic; 4 Biology Centre, Institute of Parasitology, Academy of Sciences of the Czech Republic, Ceske Budejovice, Czech Republic; 5 Department of Anthropology, Durham University, Durham, United Kingdom; 6 World Wildlife Foundation (WWF), Dzanga Sangha Protected Areas, Bangui, Central African Republic; 7 Liberec Zoo, Liberec, Czech Republic; 8 Institute of Vertebrate Biology, Academy of Sciences of the Czech Republic, Brno, Czech Republic; University of Melbourne, Australia

## Abstract

**Background:**

Hookworms are important pathogens of humans. To date, *Necator americanus* is the sole, known species of the genus *Necator* infecting humans. In contrast, several *Necator* species have been described in African great apes and other primates. It has not yet been determined whether primate-originating *Necator* species are also parasitic in humans.

**Methodology/Principal Findings:**

The infective larvae of *Necator* spp. were developed using modified Harada-Mori filter-paper cultures from faeces of humans and great apes inhabiting Dzanga-Sangha Protected Areas, Central African Republic. The first and second internal transcribed spacers (ITS-1 and ITS-2) of nuclear ribosomal DNA and partial cytochrome *c* oxidase subunit 1 (*cox1*) gene of mtDNA obtained from the hookworm larvae were sequenced and compared. Three sequence types (I–III) were recognized in the ITS region, and 34 *cox1* haplotypes represented three phylogenetic groups (A–C). The combinations determined were I-A, II-B, II-C, III-B and III-C. Combination I-A, corresponding to *N. americanus*, was demonstrated in humans and western lowland gorillas; II-B and II-C were observed in humans, western lowland gorillas and chimpanzees; III-B and III-C were found only in humans. Pairwise nucleotide difference in the *cox1* haplotypes between the groups was more than 8%, while the difference within each group was less than 2.1%.

**Conclusions/Significance:**

The distinctness of ITS sequence variants and high number of pairwise nucleotide differences among *cox1* variants indicate the possible presence of several species of *Necator* in both humans and great apes. We conclude that *Necator* hookworms are shared by humans and great apes co-habiting the same tropical forest ecosystems.

## Author Summary

We conducted analyses of DNA sequences obtained from the infective larvae of *Necator* spp. from humans and great apes inhabiting Dzanga-Sangha Protected Areas, Central African Republic. Three sequence types (I–III) were recognized in the in the ITS region, and 34 *cox1* haplotypes represented three phylogenetic groups (A–C). I-A, II-B, II-C, III-B, III-C combinations were determined. Combination I-A, corresponding to *Necator americanus*, was demonstrated in humans and western lowland gorillas; II-B and II-C were observed in humans (local inhabitants and researchers), western lowland gorillas and chimpanzees; III-B and III-C were found only in humans. Pairwise nucleotide difference in the *cox1* haplotypes between the groups was more than 8%, while the difference within each group was less than 2.1%, suggesting that each type represents a distinct species. This is the first molecular evidence that *Necator* species found in great apes can infect humans and *vice versa*.

## Introduction

The transmission of pathogens between free ranging primates and local human populations is an important topic, as primates may be reservoirs for several human diseases, and, equally, human pathogens can have devastating effects on endangered ape populations [1], [2]. Compared with the growing evidence regarding cross-infections of viruses and bacteria [e.g. 3, 4], very little is known about the diversity and transmission of helminths between humans and other primates [5].

Hookworm infections are among the commonest soil-transmitted helminthiases (STHs) in humans. It is estimated that the global number of infected people is ∼1.3 billion, of which 65,000 cases are fatal per year [6]. *Necator americanus* and *Ancylostoma duodenale* are the main hookworm species infecting humans, but the former is predominant in recent records [7]. Although the distribution of human hookworms is reported to be worldwide, Sub-Saharan Africa has the highest prevalence [7], [8]. Although humans are often regarded as the only natural host of *N. americanus* [9], other primates, including gorillas, chimpanzees, several Old and New World monkeys and other mammals, such as pangolins, have also been reported as hosts of this nematode [10]–[15] based on phenetic characteristics. In addition to *N. americanus*, three other *Necator* species have been described in African great apes, also based on morphological features of adult worms [14]–[19]. To date, efforts to better understand the true diversity and host specificity of primate hookworms have been complicated by difficulties in obtaining suitable material (adult worms) for identification, as the eggs in the faeces from infected hosts cannot be identified to species level using coproscopical methods. Thus, it is unknown whether cross-infection of *Necator* species occurs between co-existent humans and non-human primates, and if so, to what extent.

Conservation projects involving habituated great apes offer a unique opportunity to study disease transmission. The present study benefits from long-term monitoring efforts focused on pathogens transmitted among the gorillas and people in the natural forest ecosystem of Dzanga Sangha Protected Areas in the south-western Central African Republic. To evaluate the zoonotic potential of *Necator* spp., we studied DNA sequences originating from filariform larvae from infected humans, western lowland gorillas and chimpanzees at different stages of habituation.

## Materials and Methods

### Ethics statement

The research complied with the legal requirements of the Central African Republic and adhered to the research protocols of the Dzanga Sangha Protected Areas (DSPA). The collection of samples from humans was approved by the Anthropology Department Research Ethics and Data Protection Committee, University of Durham, U.K. Informed, verbal consent was obtained from all examined persons directly and via interpretative assistance, which was documented before proceeding with data collection. All samples, corresponding consent and data documentation were anonymized. It was not possible to obtain written consent from all human subjects, as many were illiterate. An institutional review board approved the use of oral consent. Collection of faecal samples from gorillas and chimpanzees was non-invasive and therefore did not cause distress to the animals. Sample importation to the EU was approved by the State Veterinary Authority of the Czech Republic.

### Survey area

**Figure 1:**
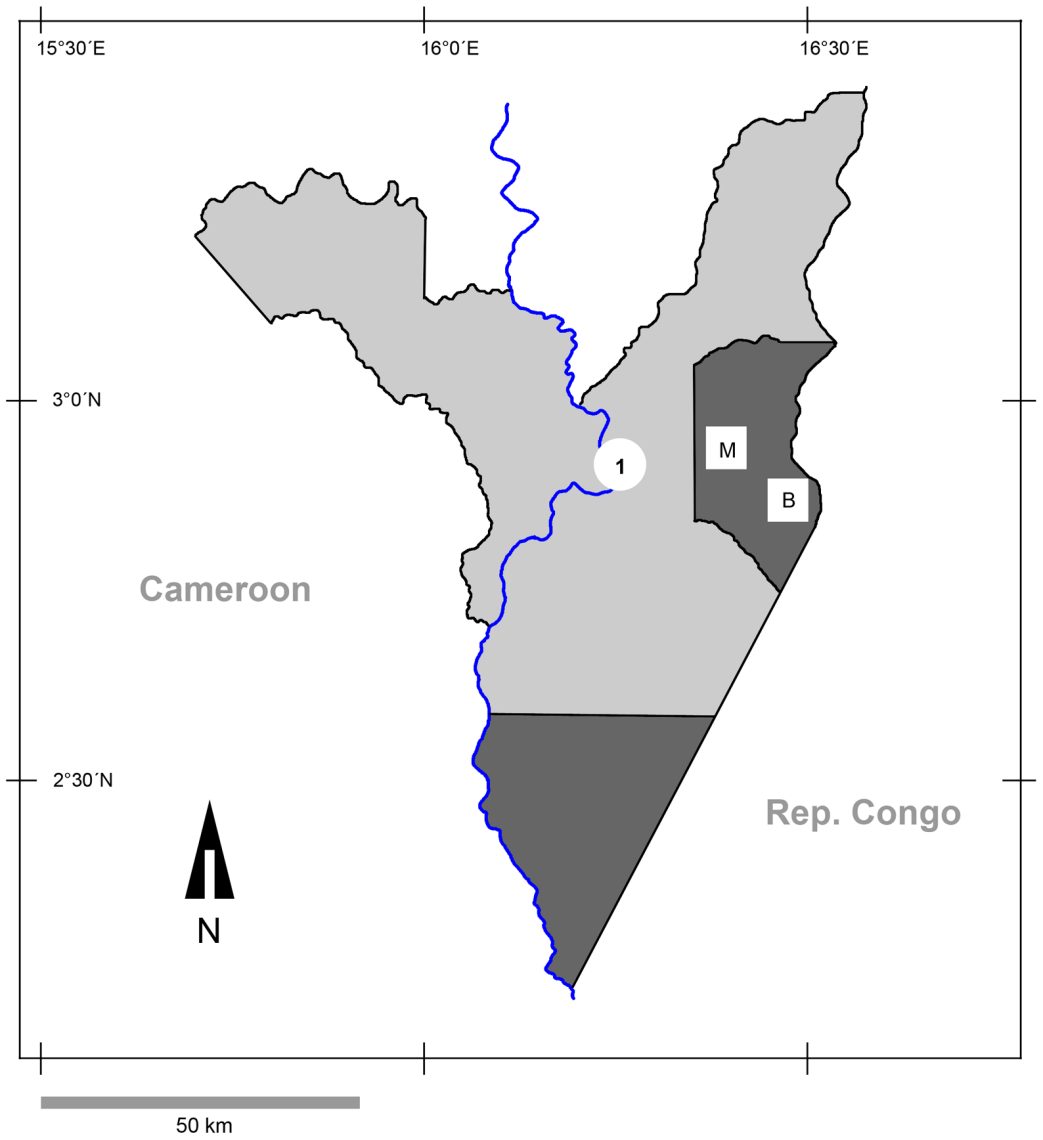
Map of study site in Dzanga-Sangha Protected Areas, Central African Republic. Dark grey – Dzanga and Ndoki Sectors of the protected Dzanga Ndoki National Park; light grey - Dzanga Sangha Dense Forest Special Reserve; 1 – location of villages Bayanga, Mossapoula and Yandumbé; M – research camp Mongambe; B – research camp Bai Hokou.

The DSPA is comprised of several zones requiring different levels of protection. For example, in the strictly protected Dzanga Ndoki National Park (1222 km^2^) human access is fully restricted, and the Dzanga Sangha Dense Forest Special Reserve (3159 km^2^) is a multiple-use zone in which human activities are differentially controlled [19] (Fig. 1). The human population density in the DSPA is low; the entire population is estimated at 7400 [20]. There are no permanent inhabitants in the Park, but many employees (e.g., gorilla trackers and assistants, ecoguards), foreign researchers and volunteers live temporarily in the research/ecoguard camps and move around the Park. The park is also visited by tourists, although they are not permitted to stay overnight. High densities of western lowland gorillas (*Gorilla gorilla gorilla*) have been reported in the DSPA, while densities of central chimpanzees (*Pan troglodytes troglodytes*) in this area are low [19]. Although gorillas and chimpanzees predominantly inhabit the National Park, they are also present to a lesser extent in the Special Reserve area [21]. In 1997, the DSPA launched the Primate Habituation Program (PHP), with the specific aim of habituating western lowland gorillas for tourism and research.

### Study subjects

Filariform larvae were raised using modified Harada-Mori filter-paper cultures [22] from gorilla faeces from two habituated groups (Makumba, Mayele), a gorilla group under habituation and several unhabituated gorilla groups, several unhabituated groups of chimpanzees and humans in 2010 and 2011. Ape sampling was carried out at the two PHP sites in the Dzanga Sector of the National Park: (i) Bai Hokou and (ii) Mongambe and their surroundings (Fig. 1). Only fresh faeces encountered during follows of habituated gorillas or those found early in the morning (7–9 am) in their night nests (unhabituated apes and gorillas under habituation) were processed in cultures. The human samples were obtained during regular health monitoring checks involving local people, including park ecoguards, the PHP trackers, project assistants and their family members. Staff family residences were outside of the Park, in the town of Bayanga, and villages of Yandoumbé and Mossapoula (Fig. 1), but all sampled humans regularly entered and stayed in the park for work. Additional samples were obtained from two European researchers (team members), who were diagnosed with hookworm infections after returning from DSPA.

After two weeks, the copro-cultures were examined using a magnifying glass, and larvae were fixed in 96% ethanol and stored in tubes. The larvae were identified to the genus level morphologically before extracting DNA [23].

### DNA extraction and enzymatic amplification

Genomic DNA was extracted from individual larvae. Each larva was transferred on to a drop of distilled water on a sterilized plastic dish, cut at mid-body with a sterilized fine needle; put in a 200 µL PCR tube containing 50 µL of liquid phase Dexpat (Takara Bio. Inc., Otsu, Shiga, Japan), heated at 100°C for 20 min, and then cooled on ice. Subsequently, 15 µl of the solution was added to the 60 µl PCR mix, which contained 1 µl of KOD-Neo polymerase (Toyobo Co., Tokyo, Japan). PCR was performed using a thermal cycler (PC-801, ASTEC Co., Ltd., Fukuoka, Japan). The primer sets for amplification and sequencing of internal transcribed regions (ITS) of *Necator* ribosomal DNA (rDNA) were Civ18S1500F 5′-TTATTTCCCTTGAACGAGGAAT-3′ (forward) and Nem5.8R 5′-TCGTTAACAACCCTGAACCAGA-3′ (reverse) for ITS-1, and Nem5.8F 5′-TACCACGAATTGCAGACGCTTA-3′ (forward) and Civ28S80R 5′-ACACCTATACGCTACATTTCTCA-3′ for ITS-2. Additional primers to amplify species-specific partial ITS-1 region of *Necator* spp. were designed: AmerF3 5′-CATTGCGTTAACATTGTATACCTGT-3′ (forward), AmerR2 5′-TTGTGTTGGCGTCCACACATATTGT-3′ (reverse) and CongF 5′-GGTTTATTCGTCGTCATTATG-3′ (forward). Partial 18S rDNA was also amplified and sequenced using primer set NCF1 5′-ACGTCTGGTTCAGGGTTGTT-3′ (forward) [24] and 18SPC 5′-ACGGGCGGTGTGTRC-3′ (reverse) [25]. The primers used for amplification and sequencing of partial mitochondrial DNA cytochrome *c* oxidase subunit 1 (*cox1*) gene were those used previously [26], [27], [28], and recently designed: StrCoxAfrF, 5′-GTGGTTTTGGTAATTGAATGGTT-3′ (forward), HkCoxMidF 5′-ACTGTTTATCCACCTTTAAGTA-3′ (forward) and JB4.5 5′-TAAAGAAAGAACATAATGAAAATG-3′ (reverse).

The PCR conditions were as follows: initial denaturation at 94°C for 2 min, followed by 20 cycles of 98°C for 10 sec, −50°C for 1 min, −68°C for 1 min, 20 cycles of 94°C for 1 min, −55°C for 1 min, −68°C for 1 min, and a post-amplification extension at 68°C for 7 min. The PCR products were mixed with EzVision Three DNA Dye (Amresco, Solon, Ohio), electrophoresed in a 1.5% agarose gel plate and detected using a UV illuminator.

### Digestion with restriction enzymes

PCR products were ethanol-precipitated and vacuum-dried. Then, products were digested for 1 h with *Alu* I or *Hinc* II according to the manufacture's protocol (Takara Bio Inc.), electrophoresed and detected as described above.

### DNA sequencing

After electrophoresis, DNA bands were extracted from the gel and purified using SUPREC-01 column (Takara Bio Inc.). Products were subjected to direct sequencing using the BigDye Terminator Cycle Sequencing Kit Version 3.1 (Applied Biosystems, Foster City, California), and a genetic analyzer ABI-PRISM 3130 (Applied Biosystems). The nucleotide sequences determined in this study were registered in the DNA Database of Japan (DDBJ, http://www.ddbj.nig.ac.jp/).

### DNA sequence alignment and phylogenetic analysis

DNA sequences of ITS were aligned using ClustalW [29]. Phylogenetic analysis was made for *cox1* nucleotide and amino acid sequences by neighbour-joining (NJ) [29] and maximum-likelihood (ML) [30] methods, using MEGA5 (v. 5.03) [31]. For NJ analysis of nucleotide and amino acid sequences, evolutionary distances were computed using the Kimura's two-parameter method and the Jones-Taylor-Thornton (JTT) matrix-based model, respectively. ML analysis was based on the best fit model of amino acid substitution. For every analysis, the bootstrap values were calculated by 1,000 replicates [32].

## Results

In total, we sequenced 140 individual larvae, including 99 larvae from humans (2 researchers, 2 ecoguards, 8 PHP trackers, 5 wives and 1 child of PHP trackers), 33 larvae from 18 gorillas (5 gorillas from the habituated Makumba group, 2 from the habituated Mayele group, 1 from a group under habituation, 9 gorillas from 4 unhabituated groups) and 8 larvae from 3 unhabituated chimpanzees from a single group. We also analysed 87 larvae by restriction enzyme digestion: 59 from gorillas (8 gorillas from Makumba group, 3 from Mayele group, 1 from a group under habituation, 1 from an unhabituated group) and 28 larvae from humans (2 researchers, 1 PHP tracker and 3 wives of PHP trackers). In total 45 larvae were studied by amplification using species-specific primers: 4 larvae from each of 10 gorillas (4 from the group under habituation, 5 from Mayele group and one from Makumba group) and 2 researchers.

### Ribosomal DNA

**Figure 2:**
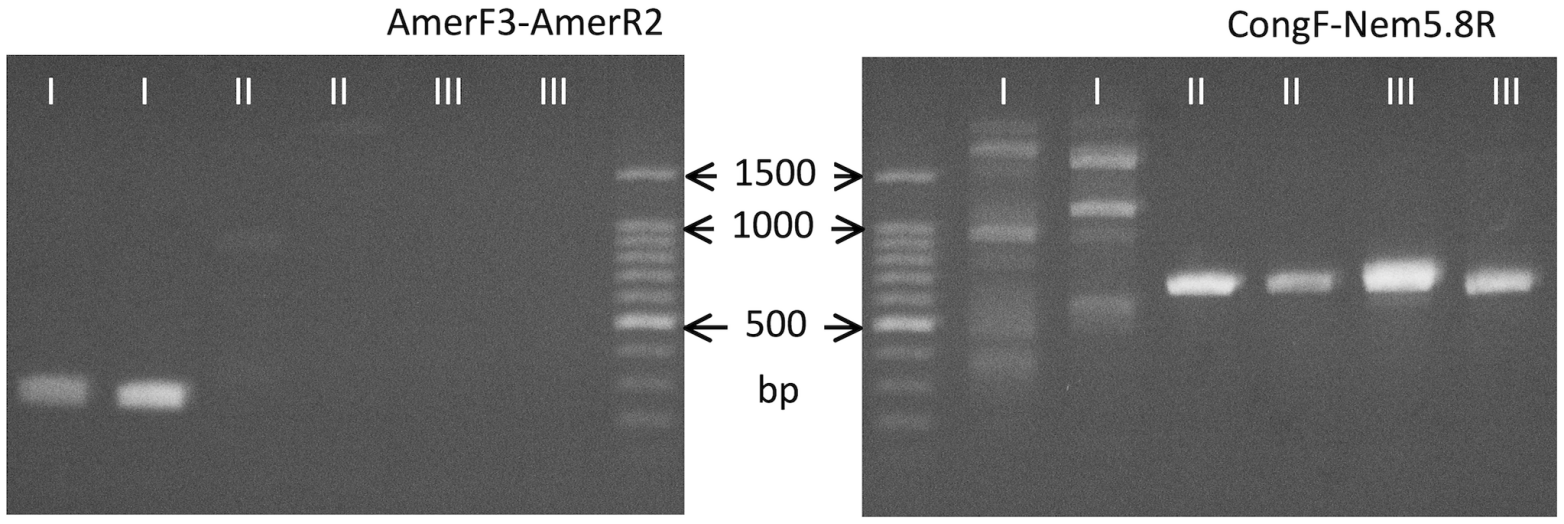
Amplification of partial ITS-1 region using specific primers. Primers AmerF3 and AmerR2 only amplified specific band of about 250-1, while no clear band was formed for types II and III. CongF and Nem5.8R resulted in formation of specific band of about 650 bp for types II and III, while prominent bands were not formed for type I.

Partial 18S rDNA, complete ITS-1, 5.8S rDNA, ITS-2 and partial 28S rDNA regions were sequenced. The primers Civ18S1500F and Nem5.8R amplified ∼900 base pairs (bp) including a 3′ region of 18S rDNA, complete ITS-1 and 5.8S rDNA regions, and primers Nem5.8F and Civ2500R amplified ∼500 bp including a 3′ region of 5.8S rDNA, the complete ITS-2 region and the 5′ region of 28S rDNA. There were three sequence types (I–III) in ITS regions, whereas other sequence regions were invariant. Type I sequence (DDBJ accession nos. AB793527, AB793528) was recorded for larvae originating from humans and gorillas; II (AB793529-AB793532) was derived from those of humans, gorillas and chimpanzees; III (AB793535, AB793536) was represented in larvae from only three people (Table 1). The primer set AmerF3-AmerR2 only amplified 223 bp of type I ITS-1, while CongF-Nem5.8R specifically amplified 663 bp of types II and III ITS-1 (Fig. 2).

**Table 1:** Prevalence of ITS sequence types (I–III) of *Necator* worms among humans and apes in Central African Republic (years 2010, 2011).*

*Necator*-positive hosts [No. examined]	Single infection			Mixed infection			
	I	II	III	I, II	I, III	II, III	I, II, III
Humans (ecoguards) [2]	2						
Humans (PHP trackers) [8]	1			5		1	1
Humans (PHP trackers' family members) [7]	5**	1		1			
Humans (European researchers) [2]				1	1		
Gorillas [14]	1	10		3			
Chimpanzees [3]		3					

*Only sequenced samples are included.

** Restriction enzyme digestion showed that two of them have mixed infection.

**Figure 3:**
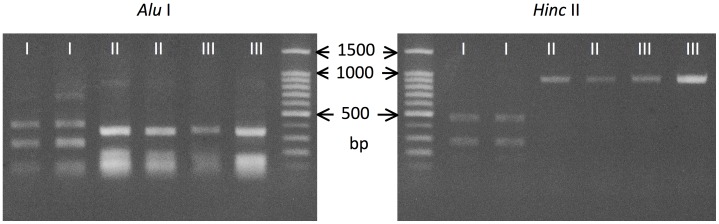
Restriction enzyme digestion of DNA sequences amplified with Civ18S1500F and Nem5.8R. By digestion with *Alu* I, two bands were formed for type I in area from 200 to 500 bp, while only one band was formed for types II and III. By digestion using *Hinc* II, two bands of about 300 and 500 bp were formed for type I, while only one band similar to original band was visible for types II and III.

ITS-1 sequence types were also distinguished by RFLP (Fig. 3). DNA sequences originally amplified with primers Civ18S1500F and Nem5.8R were subjected to digestion. Using *Alu* I, two separate bands for type I were visible in 1.5% agarose gel from 200 to 500 bp, while only one band of ∼350 bp was evident for sequence types II and III. Digestion using *Hinc* II, revealed two bands of ∼300 and ∼500 bp for type I in agarose gels, while only one band of almost the same length as the original sequence was visible when amplicons representing types II and III were digested. Using these enzymes, RFLP was not able to detect a difference between types II and III.

**Figure 4:**
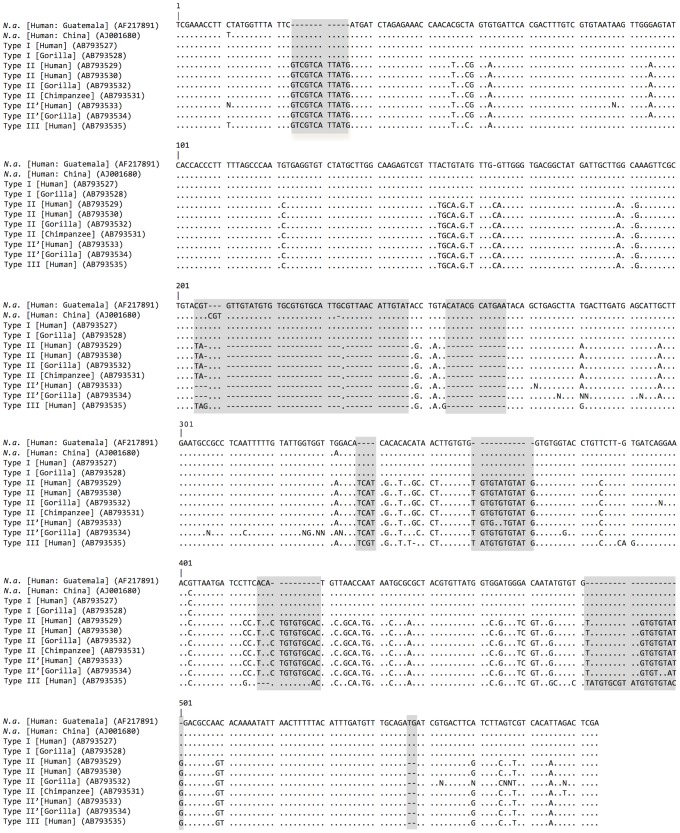
Comparison of ITS-1 sequences of hookworms from apes and humans from Dzanga Sangha Protected Areas. Host and accession number in DNA database are given in parentheses. Dots indicate homologous nucleotides with *N. americanus* (*N. a.*) from Guatemala (AF217891); dash indicates absence of nucleotide. Major indels are shaded.

**Figure 5:**
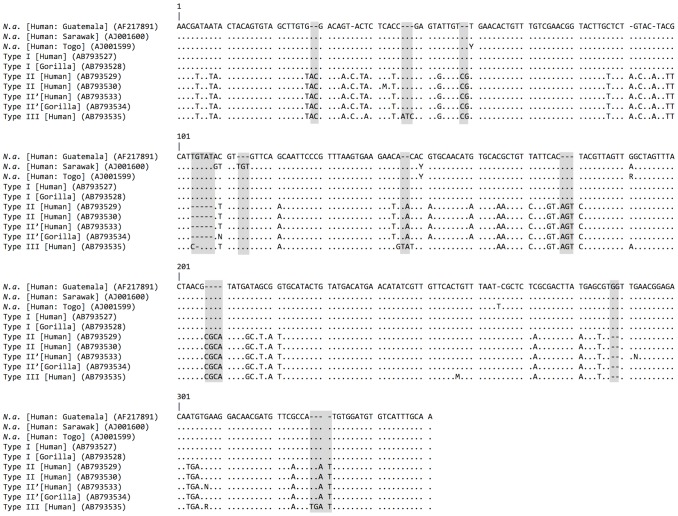
Comparison of ITS-2 sequences of hookworms from apes and humans from Dzanga Sangha Protected Areas. Host and accession number in DNA database are given in parentheses. Dots indicate homologous nucleotides with *N. americanus* (*N. a.*) from Guatemala (AF217891); dash indicates absence of nucleotide. Major indels are shaded.

Sequences of Type I (ITS-1 of 521 bp and ITS-2 of 324 bp), Type II (519 and 334 bp), and Type III (519 and 344 bp) are compared in Figures 4 and 5. Sequencing of ITS-1 in types II and III were, on occasion, ambiguous in the downstream half due to multiple repetitive elements and as a consequence typing was made on the nucleotide sequences in the upstream half of the ITS-1 region. Some sequences of type II, referred herein type II′ (AB793533, AB793534), lacked two nucleotides at two loci (Fig. 4). In these cases, the chromatograms revealed the presence of both II and II′ types in individual amplicons.

Type I was almost identical to *N. americanus* in samples from humans in Guatemala (AF217891: [33]) and Togo (AJ001599: [34]) differing only in one nucleotide substitution or insertion, respectively (Figs. 4 and 5). This type only slightly differed from *N. americanus* in a human sample from China (AJ001680; [35]) by having 3 nucleotide substitutions along with a 3-bp deletion (Fig. 4). The ITS-2 of type I sequence differed from those in samples from humans in Sarawak (AJ001600; [34]) and West Malaysia (HQ452537, HQ452539 - 459543, JF960370 - JF960403; [36]), which have a 3-bp insertion and several substitutions (Fig. 5).

Type II differs from type I by 6 indels with 10 or more consecutive bp and 55 nucleotide substitutions in ITS-1, and 11 indels each with 5 bp or less, along with 36 nucleotide substitutions in ITS-2 (Figs. 4, 5). Type III resembles type II but differs by having 2 indels with 10 or 11 bp, 4 more minor indels and 15 substitutions. Pairwise ITS distance excluding indels was 0.090 between types I and II, 0.088 between types I and III, and 0.022 between types II and III.

Concomitant infections of *Necator* with ITS types I and II were found in samples from PHP trackers, a family member, a researcher and in gorillas. Moreover, infection with the combinations of types I and III, or II and III were each detected once in samples from humans. One PHP tracker harboured three sequence types concomitantly (Table 1).

### mtDNA *cox1* gene

Partial *cox1* (670 bp) was sequenced from 58 larvae. The determined *cox1* sequences represented 34 haplotypes (17 from humans, 13 from gorillas and four from chimpanzees) (AB793537-AB793571). These haplotypes were divided into three groups (A–C) based on nucleotide composition. Haplotypes belonging to group A were detected in humans (AB 793537-AB793547) and gorillas (AB793569-AB793571); those of B were detected in humans (AB793548, AB793549, AB793562-AB793564), gorillas (AB793551-AB793558) and chimpanzees (AB793559-AB793561), and those of group C were found in humans (AB793550, AB793568), gorillas (AB793565, AB793566) and a chimpanzee (AB793567).

Group A haplotypes are very similar in sequence to that of *N. americanus* (AJ417719: in a human sample from China [37]). Pairwise nucleotide differences among the groups were ≥8% (Table 2). However, the nucleotide difference among haplotypes within each group was <2.2%. The haplotype AB793547 belonging to type A showed the largest difference (4.5–4.6%) in comparison to other haplotypes of the same group. When converted into amino acids, most of the haplotypes in group A and all in group C produced an identical sequence. Twelve haplotypes of those of group B had isoleucine at position 195, which was occupied by valine in the remaining three haplotypes of group B as well as all haplotypes in groups A and C. Additionally, seven amino acid substitutions were found sporadically (Table 3).

**Table 2:** Pairwise nucleotide difference (%) between haplotypes of mtDNA *cox1* (668 bp).

	N. a.	Group A	Group B	Group C	A. d.	A. c.
*Necator americanus* [N. a.] (AJ417719)	–					
Group A	0.6–4.5*	0.1–4.6*				
Group B	8.9–9.7	8.1–11.1	0.1–1.6			
Group C	9.1–9.6	8.2–10.2	8.7–9.6	0.4–1.0		
*Ancylostoma duodenale* [A. d.] (AJ417718)	11.2	10.2–12.0	10.3–11.1	9.6–10.0	–	
*Ancylostoma caninum* [A. c.] (NC012309)	12.9	12.3–12.7	10.3–10.9	11.2–11.5	10.3	–
*Bunostomum phlebotomum* (NC012308)	10.6	10.5–12.1	9.4–10.2	8.4–8.8	10.2	11.5

*Except one haplotype (AB793547), pairwise difference was 2.1% or lower.

**Table 3:** Amino acid substitutions found among the haplotypes of partial *cox1* gene.

Haplotype	Position in amino acid sequence					
Group	13	82	168	119	191	195
Group A (14)*	L (12)	T (13)	V (13)	T (14)	V (14)	V (14)
	M (2)	I (1)	I (1)			
Group B (16)	L (16)	T (15)	V (16)	T (15)	V (15)	V (3)
		M (1)		A (1)	I (1)	I (13)
Group C (5)	L (5)	T (5)	V (5)	T (5)	V (5)	V (5)

* No. of haplotypes.

**Figure 6:**
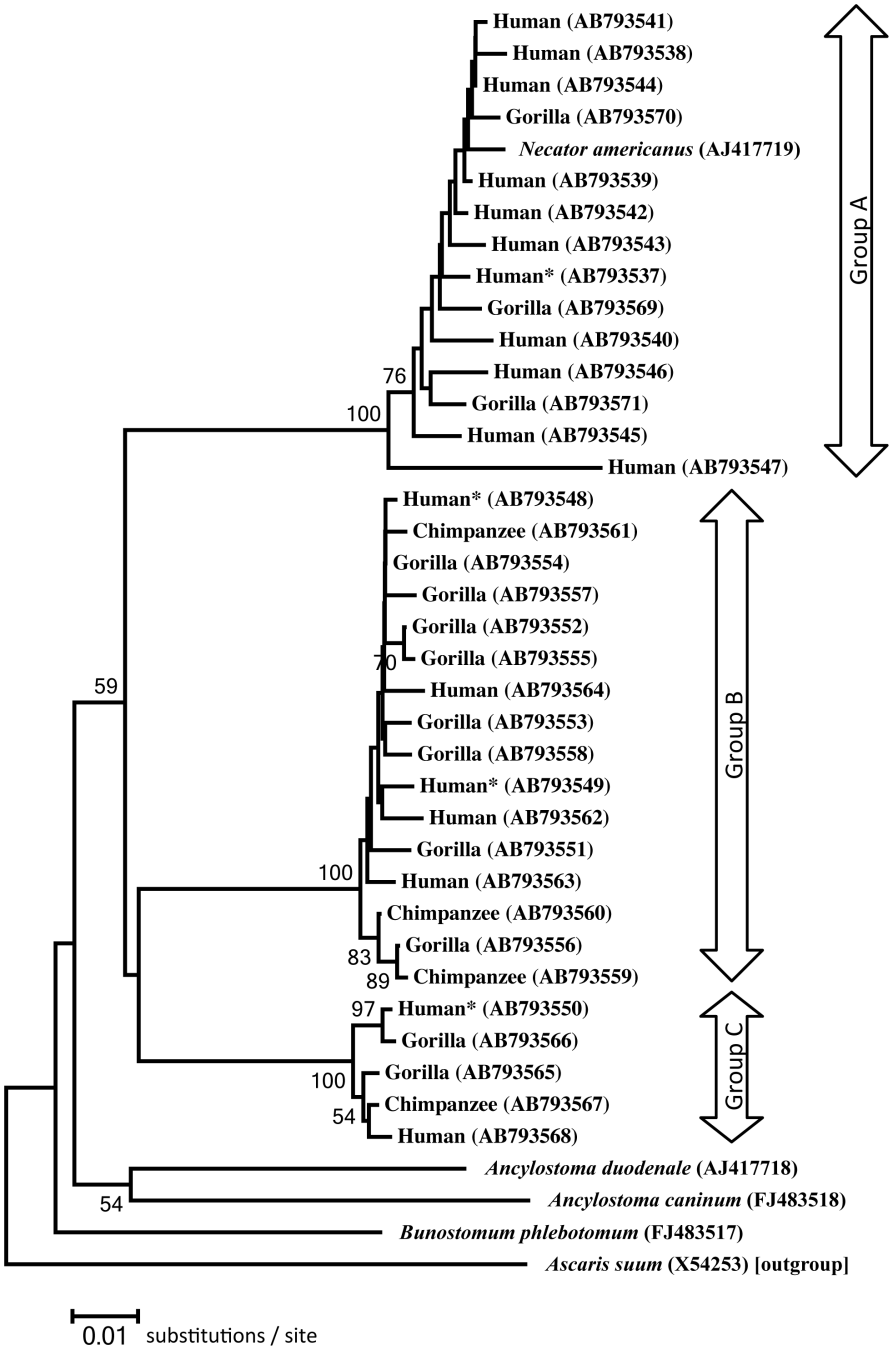
Evolutionary relationships of *Necator* spp. from apes and humans in Dzanga-Sangha Protected Areas. Tree was reconstructed by Neighbor-Joining method on mtDNA *cox1* sequences each with 669 nucleotides. Host and accession number in DNA database in parentheses are given at each branch. Humans are CAR people except for two Europeans marked with asterisks, who acquired the infection in CAR. Bootstrap values larger than 50 are shown.

Neighbour-joining reconstruction based on 669 nucleotides confirmed separation into three major groups corresponding to the haplotype groups A to C (Fig. 6). The clade of group A contained *N. americanus* (AJ417719). According to this tree, the three groups split almost the same ancestral point. Phylogenetic analysis of amino acid sequence data using NJ and ML methods did not produce trees with significance because the difference in amino acids among types was too slight.

### Relationship between ITS-1 - ITS-2 and cox1

Of the 52 hookworm larvae for which both the ITS region and *Cox1* were sequenced successfully, ITS type I was always accompanied by haplotypes of group A of *Cox1*. This combination was identified in larvae developed in human faeces, in one habituated and two unhabituated gorillas. Combinations of type II ITS with haplotype of groups B or C *cox1* were observed in hookworm larvae from trackers, their wives, habituated and unhabituated gorillas and chimpanzees. The combination of type III with *cox1* B was only found in samples from one tracker and one researcher, and type III with C was only found once in samples from a researcher.

## Discussion

Growing human populations, habitat encroachment and fragmentation, tourism and research have led to increased levels of contact between humans and wildlife. This necessitates a better understanding of the complex nature of shared infectious diseases, as recently advocated by the One Health concept (https://www.onehealthcommission.org/). The application of molecular-based tools in the field of epidemiology [38] has significantly contributed to the understanding of zoonotic transmission of soil-transmitted nematodes between human and primates [5], [34]–[36].

Our study addresses the transmission of *Necator* between humans and great apes inhabiting the forest ecosystem on the northern edge of the Congo basin. Analyses of ITS and *cox1* sequences derived from hookworm larvae revealed a remarkable level of diversity, possibly suggesting the existence of more species of *Necator* in examined hosts than previously thought. The ITS type I was always accompanied by *cox1* of group A and both conform to indexed sequences of *N. americanus*, allowing us to identify this hookworm as *N. americanus*. The ITS sequences of type I ( = *N. americanus*) were much closer to those reported in samples from human in Guatemala than those in samples from humans in China or Malaysia [33]–[36]. This close relationship is not unexpected, as *N. americanus* in the Americas might have been introduced from Africa by human migration in the early modern ages [14]. It is remarkable that combinations of type I with *cox1* of groups B or C, and II or III with group A were not identified in the present study, although concomitant infections with up to three possible combinations were common. Multiple ITS types in a single larva were not confirmed in chromatograms, except for types II and II′. Moreover, there was no evidence of possible hybridization between groups A ( = *N. americanus*) and B or C. The pairwise nucleotide differences of *cox1* between haplotypes belonging different groups almost correspond with interspecific differences [39].

We propose that the worms with *cox1* of groups B or C (and ITS types II or III) are species that are distinct from *N. americanus*. However, species distinctiveness between *cox1* groups B and C remains unsolved. Regardless of their accompanying ITS types (namely II or III), phylogenetic analysis of *cox1* sequences showed that the taxonomic point of separation of groups B and C from each other occurred at a comparable point to that of group A ( = *N. americanus*) from groups B and C. This curious incongruity between ITS and *cox1* types/groups may be due to initial host-species geographical or within-species habitat segregation and later re-convergence, which might allow the observed divergence of *cox1*.

We conclude that the larvae, which were not consistent with *N. americanus* may in fact, correspond to other hookworms previously described in the great apes, i.e., *N. congolensis*, *N. exilidens* or *N. gorillae* [14]–[19]. However, the necessary molecular data required to confirm accurate species identification are not available, and no adult worms were available in the current study. Obtaining the sequences for the type series (if available) would help solve this taxonomic problem.

Although *N. americanus* has been recorded previously in gorillas, chimpanzees and other non-human primates [10]–[13], these records were based only on morphological identifications. Our results provide the first molecular evidence that *N. americanus* parasitizes wild western lowland gorillas, but at a much lower prevalence than we reported in humans. The design of our study allowed us to survey the hookworms in groups of gorillas with various levels of direct contact with humans. The presence of *N. americanus* in both habituated and unhabituated gorilla groups suggests that spatial co-existence in a shared forest environment is an important determinant of hookworm infection, perhaps more so than direct ape-human contact during habituation.

This is the first study to report identification of other *Necator* species than *N. americanus* in humans. It is noteworthy that the genotypes other than those known for *N. americanus* are seemingly highly prevalent in people with close contact with wild gorillas, namely the gorilla PHP trackers and researchers. Additionally, family members of gorilla trackers were also infected with *Necator* species other than *N. americanus*, but to a lesser degree. We suggest that local people commonly harbour *N. americanus*, but may acquire infections of *Necator* with ITS sequences types II and III as a result of increased exposure to infection due to their life style. As traditional hunter-gatherers, they frequently enter the forest and may be exposed to various *Necator* infections. We also emphasize that as the local people often walk bare-foot, the probability of them becoming infected with filariform larvae via exposed skin contact with the ground is high. Although type III ITS sequence detected in human samples was not identified in ape samples in the current study, it has been detected in samples from a western lowland gorilla in a neighbouring country in another recent study (Hasegawa, unpublished data). We did not succeed in recovering larvae from samples from PHP assistants or their family members. The subsistence life style and hygienic conditions of PHP assistants and their families (belonging to different Bantu tribes) are different from those of the PHP trackers, as well as ecoguards, who harbour only *N. americanus*.

Our research demonstrates the potential for two-way transmission of hookworms between humans and apes in a shared forest habitat. Hypothetically, humans may bring *N. americanus* into the forest environment, resulting in infection of gorillas, and, whilst in the forest, humans may contract infections of other *Necator* species. As the sanitary conditions in local villages do not prevent and, indeed, may also encourage the establishment of local foci of infections of *Necator* species, the extent to which infected people can serve as maintenance hosts and further spread infections of *Necator* species other than *Necator americanus* needs further investigation. Foreign researchers and tourists tracking the gorillas are also exposed to the risk of becoming infected with *Necator* species other than *Necator americanus*, as demonstrated in the current study by two cases of hookworm infections found in samples of European researchers. Wearing closed shoes during gorilla tracking may prevent *Necator* infections.

The clinical importance of human hookworm infections and problems with differential diagnosis have resulted in the need for molecular tools. Very recently, even simple genotyping of ancylostomatid larvae, based on nested PCR targeting the ITS-2 [36], [39] revealed a high proportion of zoonotic infections caused by a canine hookworm, *Ancylostoma ceylanicum*, in rural human communities in Thailand [40], Malaysia and Laos [41]. The inability to differentiate between species of *Ancylostoma* based on egg/larvae morphology may have resulted in *A. ceylanicum* infection being overlooked in previous studies. Currently, *N. americanus* is considered as a common and broadly distributed human hookworm in Africa based on routine coproscopic methods. However, our results showed that the diversity of *Necator* in an African rural population is underestimated and other *Necator* species of zoonotic origin may be more common than previously believed. An improved understanding of the epidemiology and zoonotic potential of *Necator* species will assist in the correct identification of human hookworm infections, their sources, and thereby facilitate the development of appropriate hookworm infection prevention and control measures.
